# Molecular Characterisation, Evolution and Expression of Hypoxia-Inducible Factor in *Aurelia* sp.1

**DOI:** 10.1371/journal.pone.0100057

**Published:** 2014-06-13

**Authors:** Guoshan Wang, Zhigang Yu, Yu Zhen, Tiezhu Mi, Yan Shi, Jianyan Wang, Minxiao Wang, Song Sun

**Affiliations:** 1 College Marine Life Science, Ocean University of China, Qingdao, Shandong, P. R. China; 2 Key Laboratory of Marine Chemistry Theory and Engineering, Ministry of Education, Qingdao, Shandong, P. R. China; 3 College of Chemistry and Chemical Engineering, Ocean University of China, Qingdao, Shandong, P. R. China; 4 Key Laboratory of Marine Environment and Ecology, Ministry of Education, Qingdao, Shandong, P. R. China; 5 College of Environmental Science and Engineering, Ocean University of China, Qingdao, Shandong, P. R. China; 6 Key Laboratory of Marine Ecology and Environmental Sciences, Institute of Oceanology, Chinese Academy of Sciences, Qingdao, Shandong, P. R. China; University of Hawaii, United States of America

## Abstract

The maintenance of physiological oxygen homeostasis is mediated by hypoxia-inducible factor (HIF), a key transcriptional factor of the PHD-HIF system in all metazoans. However, the molecular evolutionary origin of this central physiological regulatory system is not well characterized. As the earliest eumetazoans, Cnidarians can be served as an interesting model for exploring the HIF system from an evolutionary perspective. We identified the complete cDNA sequence of HIF-1α (ASHIF) from the *Aurelia* sp.1, and the predicted HIF-1α protein (pASHIF) was comprised of 674 amino acids originating from 2,025 bp nucleotides. A Pairwise comparison revealed that pASHIF not only possessed conserved basic helix-loop-helix (bHLH) and Per-Arnt-Sim (PAS) domains but also contained the oxygen dependent degradation (ODD) and the C-terminal transactivation domains (C-TAD), the key domains for hypoxia regulation. As indicated by sequence analysis, the ASHIF gene contains 8 exons interrupted by 7 introns. Western blot analysis indicated that pASHIF that existed in the polyps and medusa of *Aurelia.* sp.1 was more stable for a hypoxic response than normoxia.

## Introduction

Molecular oxygen is the terminal electron acceptor in aerobic energy production for all animals. Maintaining oxygen homoeostasis is essential to satisfy an animal’s great mass and energy demands. To respond to oxygen fluctuations and maintain cellular oxygen homeostasis, a sophisticated mechanism has evolved in animals. The cornerstone of that central physiological regulatory mechanism is the hypoxia-inducible factor system (HIF system) [1].

The transcription factor termed HIF was first identified on the 3′enhancer of the erythropoietin gene in Hep3B cells [2]–[3]. Subsequent analysis revealed that HIF was also a basic component of most mammalian cells and did not solely exist in Hep3B cells under hypoxic conditions [4]. Most, if not all, oxygen-breathing species express the highly-conserved transcriptional complex HIF-1, which is a heterodimer composed of an alpha and a beta subunit [5]. The HIF-1α and the constitutively expressed subunit HIF-1β (aryl hydrocarbon nuclear translocator, ARNT) comprise the stable and active heterodimeric transcription complex with other auxiliary proteins, which regulate the expression of downstream genes [5]. HIF-1α and HIF-1β contain conserved basic helix-loop-helix (bHLH) and Per-Arnt-Sim (PAS) domains. The bHLH and PAS motifs participate in HIF heterodimer formation and specific binding to the target DNA sequence [6]. In addition to the conserved bHLH and PAS domains, HIF-1α also contains two regions that are oxygen dependent: oxygen dependent domain (ODD) and C-terminal transactivation domain (C-TAD) [7]. In mammals, as the direct sensors of cellular oxygen levels, prolyl hydroxylase domain enzymes (PHDs/EGLNs) and the asparaginyl hydroxylase factor inhibiting HIF (FIH) regulate the stability and transcriptional activity of HIF [8]. The two oxygen sensors catalyze the post-translational hydroxylation of ODD and C-TAD, respectively, under normoxia [9]–[10]. The hydroxylated protein is degraded after binding to the von Hippel Lindau protein (VHL) elongin B/C ubiquitin ligase complex [1]. PHDs/EGLNs and FIH are members of the 2-oxoglutarate and Fe(II) dependent oxygenase super family that catalyzes HIF-1α hydroxylation using molecular oxygen and 2-oxoglutarate as the substituent group [11]–[12]. However, in hypoxia, HIF-1α is stable and functional. Besides, the other homologs of components of the HIF family include HIF-2α (endothelial PAS domain protein 1), HIF-2β (ARNT2), HIF-3α (hypoxia inducible factor 3, alpha subunit) and HIF-3β (ARNT3). They are reported in some vertebrates from current research, and there seems to be no evidence showing that they exist in most invertebrates. HIF regulates many fundamental metabolic processes, including angiogenesis, erythropoiesis, glucose and iron transport, glycolysis, the tricarboxylic acid cycle, cell proliferation and apoptosis as well as specialized oxygen delivery systems in mammals [13]–[14].

The HIF pathway is likely present in all metazoans from the simplest animal, *Trichoplax adhaerens*, to higher animals, *Homo sapiens* [15]–[16]. Cnidarians that appeared in the pre-Cambrian period (600 MYA) are considered to be the oldest multi-organ phylum of animals in primary evolution [17]. Scientists have found that they are more tolerant of hypoxia than other higher marine organisms so as to flourish in parts of the world and occupy dominant niches in marine environments. For example, *Chrysaora quinquecirrha* medusae live more than 96 h at 1 mg O_2 _L^−1^, and their polyps can live and reproduce at 0.5 mg O_2 _L^−1^ [18]. So Cnidarians can be served as an interesting model for exploring the HIF system. Although genome sequencing and analysis revealed partial HIF-1α sequences in *Nematostella vectensis* (Anthozoa) and *Hydra magnipapillata* (Hydrozoa), a complete HIF-1α sequence has not been reported in Cnidarian [19]. Here, we report the complete cDNA and predicted amino acid sequence of HIF-1α from *Aurelia* sp.1 (Scyphozoan), one of the most common and widely distributed species of jellyfish in the ocean. In addition, we also present an analysis of the evolution of protein domains and genome structure as well as protein expression in two generations (e.g., planktonic and benthic generations).

## Materials and Methods

### *Aurelia* sp.1 Cultivation

*Aurelia* sp.1 (polyps and medusa) were provided by the Institute of Oceanography, Chinese Academy of Sciences (IOCAS). *Aurelia* sp.1 were fed *Artemia nauplius* and cultivated in 50-L fish tanks with filtered seawater (salinity: 33 PSU, 19°C). Two 3-L enclosed conical flasks were used for the hypoxic experiments. One flask was used as the reference group in which dissolved oxygen (DO) achieved saturation through bubbling. The other flask was used for the hypoxic group with an approximate 0.5 mg/L DO concentration provided by the bubbling of 99.9% nitrogen. Dissolved oxygen was monitored continuously to maintain experimental stability using a Model HQ30d multi-parameter meter (HACH, Beijing, China).

### Total DNA and RNA Isolation

Total DNA was isolated from approximately 100 mg of *Aurelia* sp.1 medusa using the DNeasy Blood & Tissue Kit (Qiagen, Hilden, Germany). Total *Aurelia* sp.1 RNA was extracted using the Transzol (Transgen, Beijing, China). Total DNA and RNA were characterized by agarose gel electrophoresis and spectrophotometry.

### Cloning and Sequencing of the Complete ASHIF cDNA

The partial *HIF-1α* for *Aurelia* sp.1 (*ASHIF*) nucleic acid sequence was obtained from RNA-seq (PRJNA219043) generated by IOCAS. Degenerate primers (CTF653 F0 and CTF653 R0, Table 1) were designed for confirmation of the partial *ASHIF* gene sequence. A full-length *ASHIF* transcript was obtained from the total RNA of *Aurelia* sp.1 medusa using 5′ and 3′ end RACE. For the 5′ end RACE, the cDNA was synthesized via reverse transcription (RT) using the SMARTer RACE cDNA Amplification Kit (Clontech, Mountain View, CA, U.S.A.), and the PCR amplification was performed with the CTF653 R6 and CTF653 R5 primers (Table 1) using Clontech Advantage PCR Kit (Clontech, Mountain View, CA, U.S.A.). Similarly, the cDNA from the 3′ end RACE was synthesized by RT using the 3′-Full RACE Core Set with PrimeScript RTase (Takara, Dalian, China), and the PCR amplification was performed with the CTF653 F2 and CTF653 F3 primers (Table 1) using TaKaRa LA Taq with GC Buffer (Takara, Dalian, China). All products from the 3′ end and 5′ end RACE PCRs were gel-purified using the TaKaRa MiniBEST Agarose Gel DNA Extraction Kit (Takara, Dalian, China) and cloned into *Escherichia coli* using a T-Vector pMD (Takara, Dalian, China). Three positive clones were randomly selected for each gene product and sequenced.

**Table 1:** Primers used to obtain the complete cDNA sequence of *ASHIF.*

Primer name	Nucleotide sequences (5′-3′)
**CTF653 F0**	ACATTTGCAAAAAGCACACAAG
**CTF653 R0**	GGCTGTTATTTGCGTTTCCAAAATCG
**CTF653 F2**	AACGAGCCCACGAGACATCCAATA
**CTF653 F3**	GGGAGGCTGGATTTGGATGCTGAC
**CTF653 R5**	GTCTTGGGAATGATAGGCAACTGGCT
**CTF653 R6**	AACCCATCTAAGGCATCCATTATTGAC

### Supplementing the *N. vectensis HIF-1α* Nucleic Acid Sequence

To study the evolution of HIF-1α in Cnidarians, we searched the genomic sequence of *N. vectensis* to supplement its *HIF-1α* gene (*NVHIF*) and predicted protein sequence of *NV*HIF (p*NV*HIF) based on the partial *NVHIF* nucleic acid sequence (XM_001637871) in GenBank.

### Analysis of Predicted HIF-1α Protein (p*AS*HIF) and p*NV*HIF

We determined the open reading frame (ORF) in the *ASHIF* and *NVHIF* gene from the complete cDNA sequence, which were translated into their respective amino acid sequence with universal code using the DNAMAN 7.0 software. The analysis of the basic properties (molecular weight, isoelectric point, transmembrane helix predictions and hydrophobicity or hydrophilicity profile) were performed using DNAMAN 7.0, TMHMM Server v. 2.0 (http://www.cbs.dtu.dk/services/TMHMM-2.0/) and the Protscale program (http://web.expasy.org/protscale/). Multiple sequence alignments with other metazoans were performed to elucidate the functional domain of p*AS*HIF and p*NV*HIF.

### Phylogenetic Analysis

Multiple sequence alignments of the deduced *AS*HIF amino acid sequences were performed with Clustal X, and the default parameters setting was used. The sequences included the following items: *T. adhaerens* (AFM37575), *H. magnipapillata* (XP_002167197), *N. vectensis*, *Ascaris suumgi* (BAJ17131), *Caenorhabditis elegans* (CAA19521 and CAB07380), *Palaemonetes pugio* (AAT72404), *Litopenaeus vannamei* (FJ807918), *Metacarcinus magister* (ABF83561), *Drosophila melanogaster* (AAC47303), *Apis mellifera* (XP_392382), *Tribolium castaneum* (XP_967427), *Strongylocentrotus purpuratus* (XP_783102), *Hemiscyllium ocellatum* (ABY86627), *Mustelus canis* (ABY86628), *Megalobrama amblycephala* (ADF50043), *Hypophthalmichthys molitrix* (ADJ67806), *Danio rerio* (NP_956527, ABD33838 and AAQ94179), *Xenopus laevi* (NP_001080449), *Gallus* (NP_989628), *Oryctolagus cuniculus* (NP_001076251), *Sus scrofa* (NP_001116596), *Bos Taurus* (NP_776764), *Mus musculusgi* (AAH26139, NP_034267 and AAC72734), and *H. sapiens* (NP_001521, NP_851397, NP_001230013, AAC51212 and AC007193). The maximum likelihood method using the Jones-Taylor-Thornton matrix was applied to the molecular phylogenetic analysis in the Mega 5.0 program [1]. The reliability of the estimated tree was evaluated using the bootstrap method with 1,000 replications.

### Genomic Structure of *ASHIF*

Six pairs of degenerate primers (AAHIF-1F and AAHIF-1R; AAHIF-2F and AAHIF-2R; AAHIF-3F and AAHIF-3R; AAHIF-4F and AAHIF-4R; AAHIF-5F and AAHIF-5R; AAHIF-6F and AAHIF-6R, Table 2) were designed against the complete *ASHIF* cDNA sequence to clone genome sequence. The fragment genome sequences were obtained by PCR using TransTag-T DNA polymerase (Transgen, Beijing, China) according to the manufacturer’s recommendations. The PCR was performed in a 25-µL final volume reaction including 2.5 µL of 10×TransTag-T buffer, 2 µL of dNTPs, 1 µL of DNA from medusa, 1 µL (10 µM) of each primer, 0.5 µL of TransTag-T polymerase and 17 µL of ddH_2_O. The annealing temperature was 52°C for the following degenerate primers: AAHIF-1, AAHIF-2, AAHIF-4 and AAHIF-5. An annealing temperature of 54°C was used for AAHIF-3. Finally, all PCR products were directly sequenced using their own degenerate primers.

**Table 2:** Primers used to obtain the genome sequence of *ASHIF.*

Primer name	Nucleotide sequences (5′-3′)
**AAHIF-1F**	GGAAAAATGTGAGCGAAATAGG
**AAHIF-1R**	TGGGAATGATAGGCAACTGG
**AAHIF-2F**	AGC CAG TTG CCT ATC ATT CC
**AAHIF-2R**	ATT CCA AGA TAC TGT GCC
**AAHIF-3F**	GATGCCTTAGATGGGTTCA
**AAHIF-3R**	CTCGCAAGTCCAGTGTATGT
**AAHIF-4F**	AACACATACACTGGACTTGCGA
**AAHIF-4R**	ATTTGACAGCCTCACCGTTT
**AAHIF-5F**	GCCCACGAGACATCCAATAA
**AAHIF-5R**	AGTAGCGGGTCCGATAGCC
**AAHIF-6F**	CATTTGACGACTTGCCAGATTC
**AAHIF-6R**	ATACCTTGTTTATACGGCGACA

### Evolutional Analysis of the HIF-1α Genomic Structure

To identify the *HIF* genomic structure evolution for animals, we aligned the cDNA sequences of *HIF* to the genomic sequences using the SIM4 software (http://pbil.univ-lyon1.fr/sim4.php). The accession numbers in GenBank for cDNA and genomic sequences are listed in Table 3.

**Table 3:** The accession numbers in GenBank for *HIF* nucleic acid sequences in alignment.

Species	Accession for cDNA	Accession for genome
*Trichoplax adhaerens*	JQ844128.1	JGI: 56360*
*Hydra magnipapillata*	XM_002167161	NW_004167682
*Aurelia* sp.1	KJ411880	KJ411883
*Nematostella vectensis*	XM_001637871	NW_001834388
*Strongylocentrotus purpuratus*	XM_778009.3	NW_003577559
*Homo sapiens*	NM_001530.3	NG_029606.1

*The sequence came from DOE Joint Genome Institute.

### Western Blot Analysis

**Figure 1:**
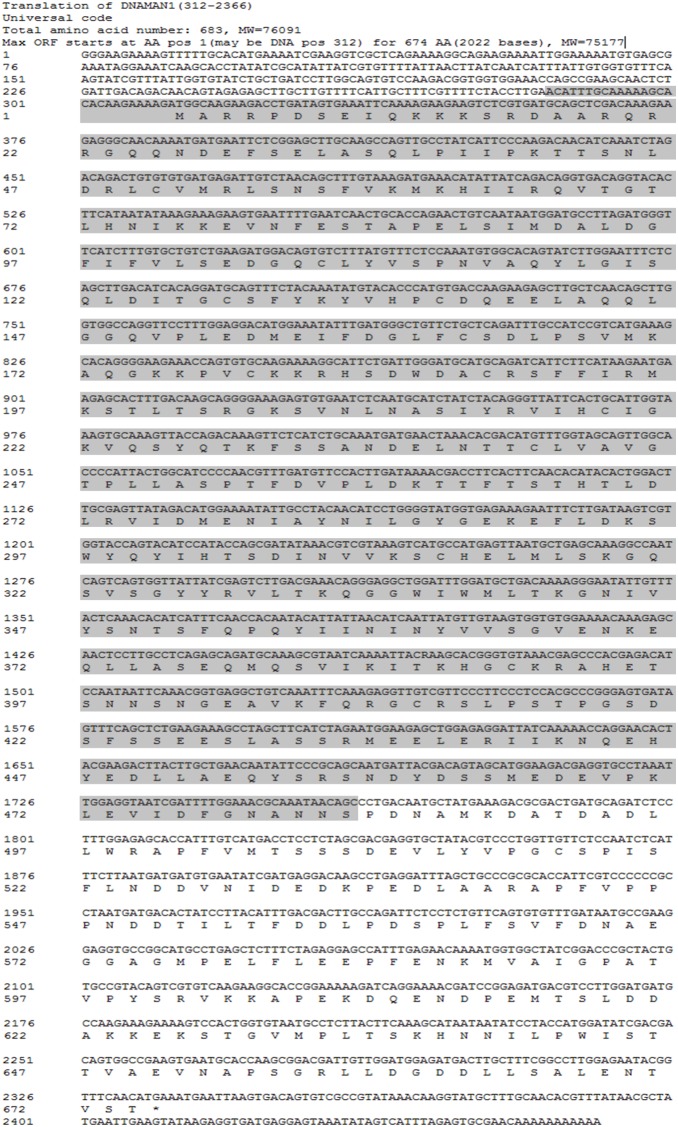
Nucleotide and predicted amino acid sequence of HIF-1α in *Aurelia* sp.1. The asterisk indicated the stop codon, and the shadow portion was used as the recombinant fragment to generate the polyclonal antibody.

The polyps and medusa grew in the control and hypoxia (DO ∼0.5 mg/L) conditions for 18 hours. Total proteins were extracted from the polyps and medusa using Transzol (Transgen, Beijing, China) and then were analyzed by immunoblotting following SDS-PAGE separation. The rabbit anti-p*AS*HIF polyclonal antibody (PA) was purified from rabbit serum after immunisation with the *AS*HIF recombinant fragment, which expressed in vitro in BL21 *E.coli* using the Pet-32a vector. The recombinant fragment contains 492 residues from the part of full-length *ASHIF* nucleic acid sequences (284 to 1760 bp, the shaded portion of Fig. 1). And we defined the recombinant fragment protein as the positive control in the Western blotting assay. The Rabbit anti-p*AS*HIF PA was used at a 1∶1,500 dilution, and the reference anti-beta-actin (Bioss, Beijing, China) PA was also used at a 1∶1,500 dilution. The HRP-conjugated goat anti-rabbit IgG monoclonal secondary antibody (Transgen, Beijing, China) was used at a dilution of 1∶5,000. The analysis of densitometry was performed using Quantity One software.

## Results

### *Aurelia* sp.1 *HIF-1α* Coding Sequence

**Figure 2:**
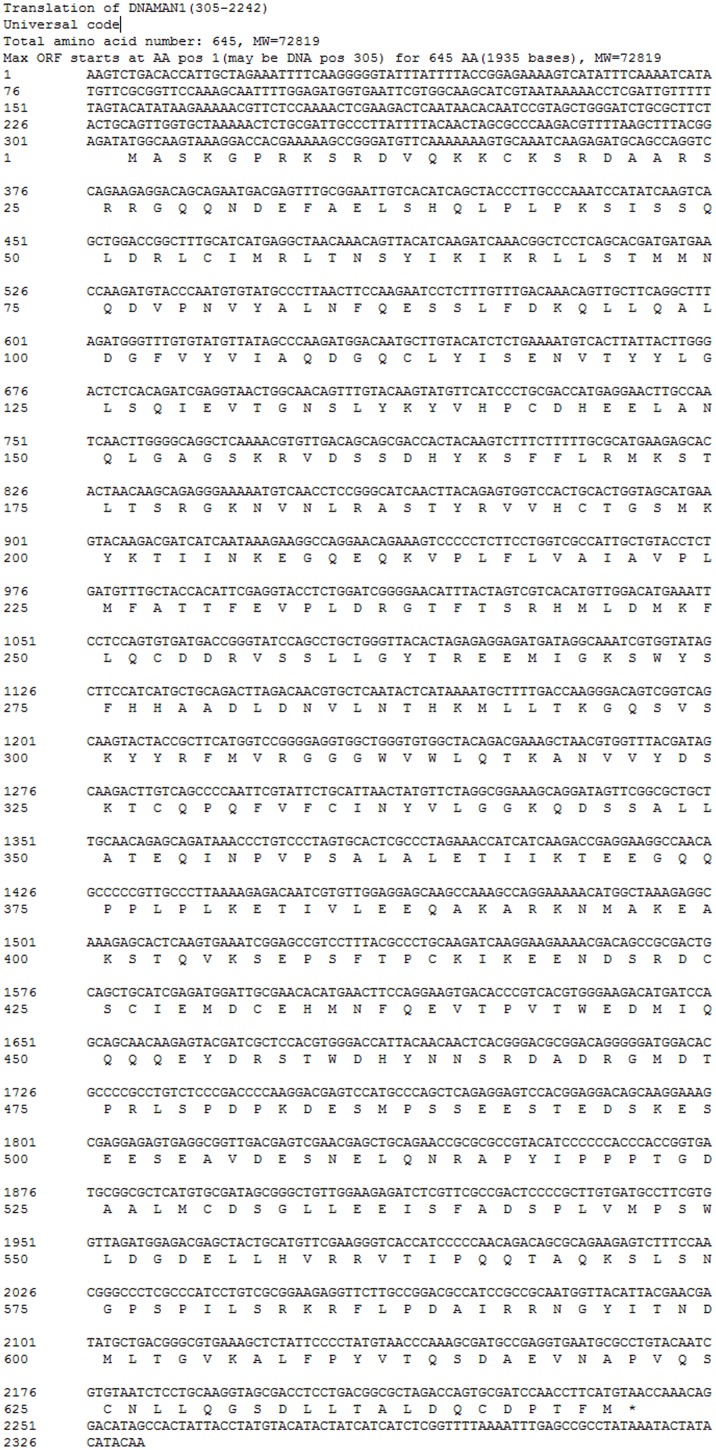
Predicted nucleotide and amino acid sequence of HIF-1α in *N. vectensis*. The asterisk indicated the stop codon.

**Figure 3:**
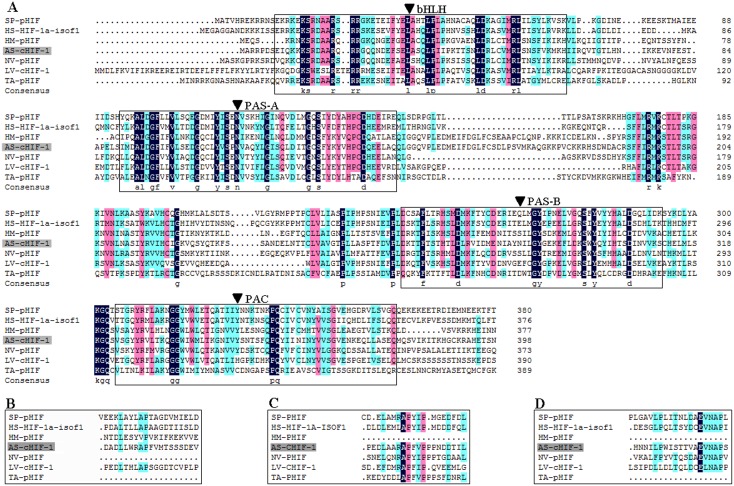
Alignment of HIF-1α deduced amino acid sequences with *T. adhaerens*, *H. magnipapillata*, *N. vectensis*, *A* sp.1, *L. vannamei*, *S. purpuratus* and *H. sapiens* homologues. (A) The functional domains that are included as solid boxes contain bHLH and PAS domain. (B) The N-ODD domains. (C) The C-ODD domains. (D) The C-TAD domains.

The annotated RNA-seq result revealed that the partial *ASHIF* sequence was aligned to the HIF-1α of *Hydra vulgaris* (XP_002167197) with blasting to GenBank protein database. Based on the partial *ASHIF* nucleic acid sequence, we obtained 284 bp and 694 bp fragments of *ASHIF* using 5′ end and 3′ end RACE, respectively. The full-length *ASHIF* cDNA sequence was 2,466 bp. The sequence was comprised of a 2,025 bp ORF, a 311 bp 5′UTR and a 130 bp 3′UTR that included a portion of the ploy-A tail after assembling (Fig. 1). The predicted *AS*HIF protein (p*AS*HIF) contains 674 residues with a calculated molecular weight (MW) of 75,177 D and an isoelectric point (pI) of 4.70. In addition, we searched full-length *N. vectensis HIF* (*NVHIF*) genomic sequence based on the partial *NVHIF* nucleic acid sequence (XM_001637871) in GenBank and obtained the remaining 1,009 bp fragment. The full-length *NVHIF* cDNA sequence was 2,332 bp. The sequence was comprised of a 1,938 bp ORF, a 304 bp 5′UTR and a 90 bp 3′UTR that excluded a portion of the ploy-A tail after assembly (Fig. 2). The predicted *NV*HIF protein (p*NV*HIF) contains 645 residues with a calculated MW of 72,819 D and a pI of 5.70. The analysis using the TMHMM Server v. 2.0 indicated that no transmembrane helices were present in the p*AS*HIF and p*NV*HIF sequences. The p*AS*HIF and p*NV*HIF sequences displayed a hydrophilic profile using the Protscale program. The p*AS*HIF displayed similarities with *T. adhaerens* (30%), *H. magnipapillata* (45%), *N. vectensis* (35%), *L. vannamei* (32%), *S. purpuratus* (33%) and *H. sapiens* (34%). The p*AS*HIF contains complete HIF-1α features including the bHLH, PAS, N-ODD, C-ODD and C-TAD domains (Fig. 3). However, the p*NV*HIF sequence possesses all of the characteristic domains with the exception of the N-ODD domain. The conserved bHLH domain is comprised of residues 7 to 63 in p*AS*HIF and residues 11 to 67 in p*NV*HIF. The PAS-A, PAS-B and PAC regions comprise the PAS domain which is composed of residues 89 to 678 and 94 to 353 in p*AS*HIF and p*NV*HIF, respectively. The conserved proline (pro) hydroxylated by PHDs is located in the N-ODD (residue 501) and C-ODD (515) of p*AS*HIF, and the asparagine (Asp, residue 649) hydroxylated by FIH is located in the C-TAD. Similarly, the conserved proline is located in the C-ODD (515) of p*NV*HIF, and the asparagine (616) is located in the C-TAD.

### Phylogenetic Analysis of HIF-1α Protein

**Figure 4:**
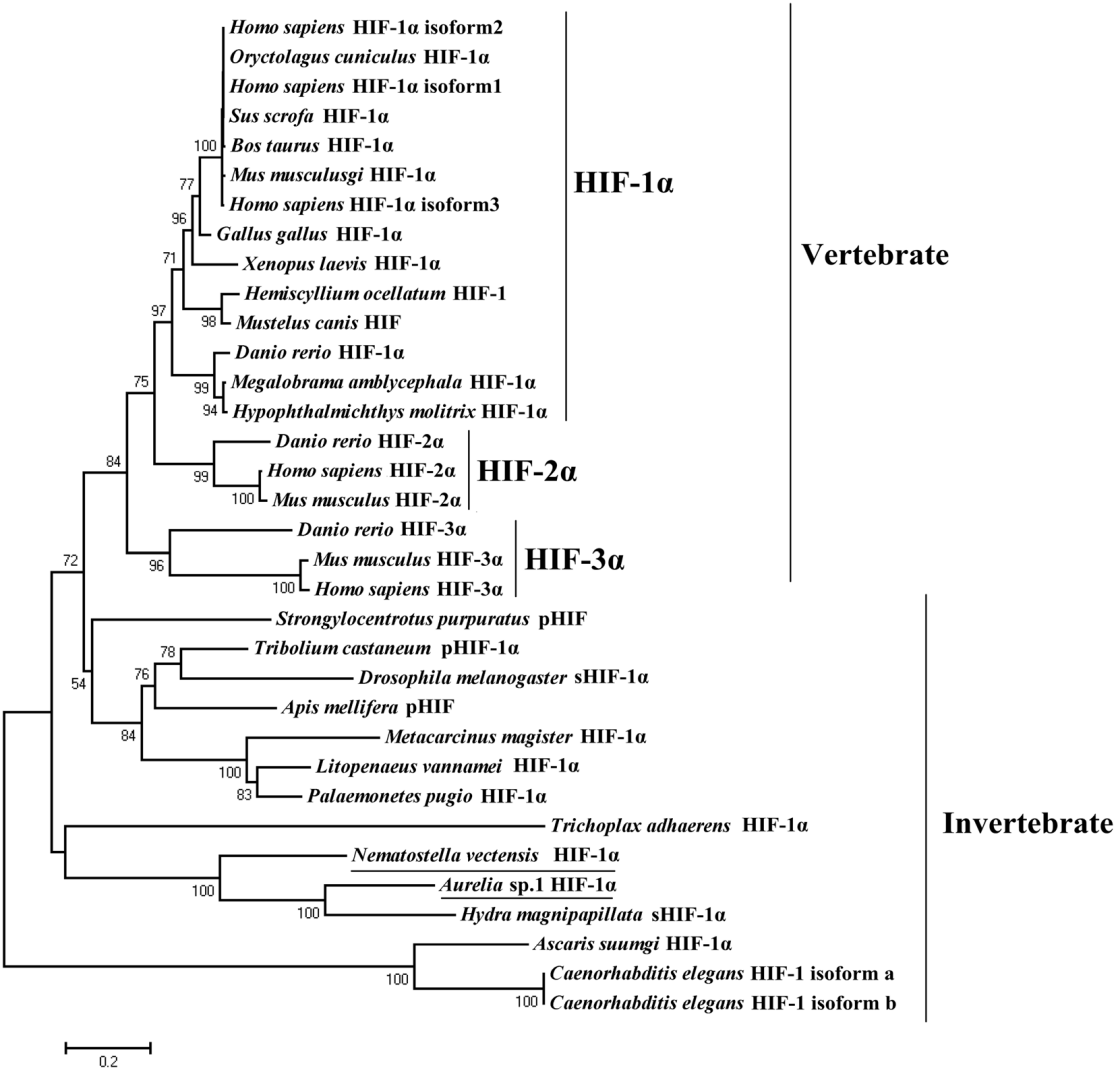
Maximum likelihood phylogenetic tree based on protein sequences of HIF. The numbers at the nodes represented the credible values that were calculated from 1000-times bootstrap tests. The underline represented the species that we obtained the full-length sequence.

The phylogenetic tree of HIF indicates that the vertebrate clade separated from the invertebrate clade after *S. purpuratus* (Fig. 4). The vertebrate clade contains three groups: HIF-1α, HIF-2α and HIF-3α. The invertebrate clade was divided into four subclades: 1. Placozoa (*T. adhaerens*); 2. Cnidaria (*H. magnipapillata*, *Aurelia* sp.1 and *N. vectensis*); 3. Nematoda (*A. suumgi* and *C. elegans*); 4. Echinodermata (*S. purpuratus*) and Arthropoda (Insecta and Crustacea). Specifically, *N. vectensis* was separated from *H. magnipapillata* and *Aurelia* sp.1 with 100% bootstrap support in the Cnidarian subclade.

### *ASHIF* Genomic Sequence

According to the cDNA sequence, 6 pairs of degenerate primers (Table 2) were designed to obtain 6 amplification fragments (2,167 bp, 1,283 bp, 2,226 bp, 4,047 bp, 612 and 404 bp) from total DNA. Six overlapping sequences were assembled after sequencing. Finally, we confirmed that the length of *ASHIF* genomic sequence was 10739 bp and the *ASHIF* coding sequence contained 8 exons interrupted by 7 introns.

### Evolution of HIF-1α Functional Domain and Genomic Structure

**Figure 5:**
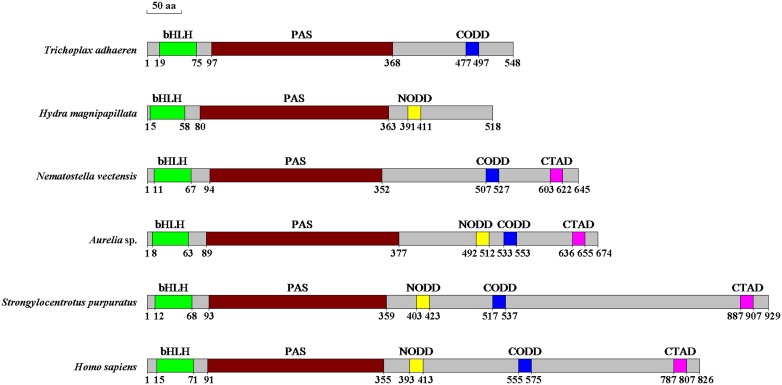
The protein domain structure alignment of HIF-1α with *T. adhaerens*, *H. magnipapillata*, *N. vectensis*, *Aurelia* sp.1, *S. purpuratus* and *H. sapiens*.

**Figure 6:**
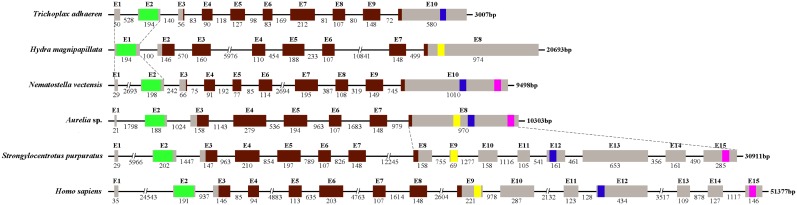
The genomic structure, length and organization of *HIF-1α* from *T. adhaerens*, *H. magnipapillata*, *N. vectensis*, *Aurelia* sp.1, *S. purpuratus* and *H. sapiens*. Boxes represented the sequences of exons of the gene and thin lines indicated the sequences of introns. Values above the lines and the boxes indicated the sizes of the introns and exons, respectively. “E1 to E15” represented “Exon1 to Exon15”. The exons included bHLH domain (highlighted in green), PAS domain (highlighted in red), N-ODD domain (highlighted in yellow), C-ODD domain (highlighted in blue), and C-TAD domain (highlighted in pink).

The HIF-1α protein sequences with functional domain annotation from *T. adhaerens* to *Homo sapiens* were listed to investigate the evolution differences (Fig. 5). The *H. magnipapillata* polypeptide (518 residues) was the shortest in length, whereas the *S. purpuratus* (929 residues) was the longest. The HIF-1α proteins from the various species did not possess all characteristic functional domains. For instance, the simplest animal *T. adhaerens* did not possess N-ODD and C-TAD domains. *H. agnipapillata* did not contain C-ODD and C-TAD domains, and *N. vectensis* lacked the N-ODD domain. Genomic structure analysis indicated that *AS*HIF and the *HIF-1α* gene from *H. magnipapillata* (*HM*HIF) consisted of 8 exons interrupted by 7 introns, whereas *NV*HIF and the *HIF-1α* in *T. adhaerens* (*TA*HIF) contained 10 exons interrupted by 9 introns (Fig. 6). It was worth noting that the *HIF-1α* genomic sequence in higher animals (*S. purpuratus* and *H. sapiens*) consisted of 15 exons and 14 introns. Sequence comparison demonstrated that exon 1 and 2 from other species were combined to form exon 1 in *H. magnipapillata*. The last exon in the lower organisms (Placozoa and Cnidaria) differentiated into 8 exons in *S. purpuratus* and 7 exons in *H. sapiens*. The differences in the corresponding introns in all six species resulted in the substantial differences in the *HIF-1α* genome sequence length.

### p*AS*HIF Expression in Polyps and Medusa under Hypoxia

**Figure 7:**
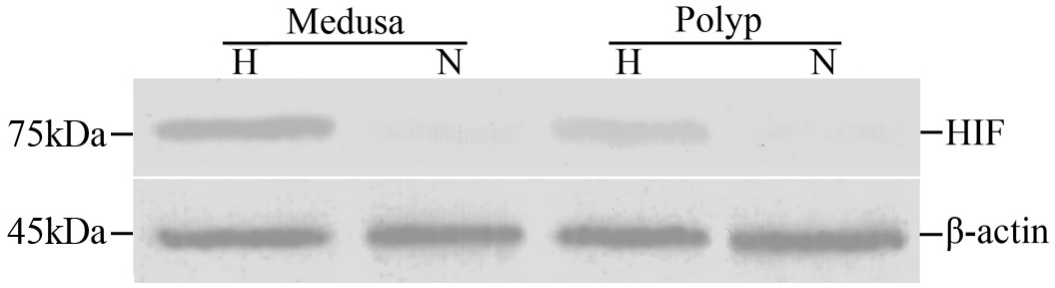
Expression of p*AS*HIF protein in polyps and medusa. The *AS*HIF recombinant fragment protein was used as the positive control, and the actin protein was used as the standard to determine the quantity of the total protein. M represented protein marker, N represented reference group (normoxia condition), H represented hypoxic group and P was positive control.

**Figure 8:**
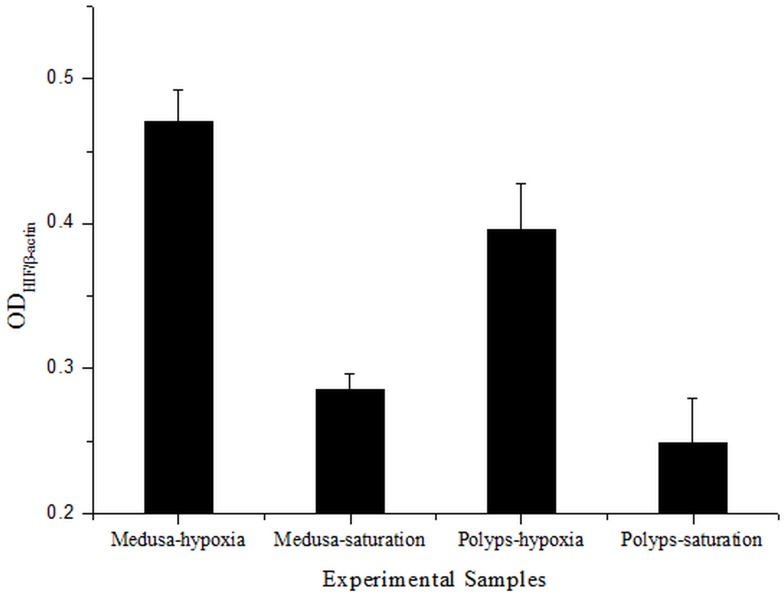
The densitometry analysis of protein expression. The vertical axis represented the densitometry ratio of protein HIF and β-actin, and the horizontal axis represented the different samples of Western blot assay.

There was a visible single band with a molecular mass of approximately 75 kDa in the polyps and medusa under hypoxia conditions (DO ∼0.5 mg/L) while a looming band under control (DO ∼8.0 mg/L) after 18 hours cultivation (Fig. 7). Densitometry analysis of the Western blot verified that the relative expression of p*AS*HIF was highest in the medusa under hypoxia, followed by hypoxic polyps, and lowest in polyps under control (Fig. 8). A protein band of approximately 45 kDa was identified as the beta-actin reference protein.

## Discussion

Jellyfish have developed a strong ability to adapt to environmental changes, particularly to tolerate seasonal hypoxia through million years of evolution [17]–[18]. Although the PHD-HIF system potentially plays an important role in this process, information regarding HIF-1α in jellyfish has not been reported. Loenarz and his colleagues found evidence for a functional HIF system in the simplest animal *T. adhaerens* [16]. In addition, similar bHLH-PAS transcription factors were identified in *N. vectensis* by genome analysis [15]. Furthermore, we supplemented the *NVHIF* sequence by searching the genome sequence. Simultaneously, we obtained partial *HIF-1α* for *Aurelia* sp.1 (*ASHIF*) nucleic acid sequence from RNA-seq. A homolog of HIF-1β, some PHDs and FIH analogues including PHD3, PHD4, asparaginyl beta-hydroxylase-like and VHL were also verified in *Aurelia* sp.1 by the annotation. After that, the full-length *ASHIF* sequence was assembled using RACE, which was the first report of the *HIF-1α* complement in Scyphozoan (jellyfish). The typical HIF-1α protein domains of p*AS*HIF and p*NV*HIF include the bHLH and PAS, which mediate protein interactions with other proteins or DNA, as well as ODD and C-TAD domains, which control HIF-1α degradation under normoxic conditions by post-translational hydroxylation of conserved proline and asparagine residues (Fig. 3). The N-ODD proline residues in *Aurelia* sp.1 are located in the LXXLAP region (from residues 495 to 501) that is conserved in all animals. The second proline residues of the C-ODD occurred in the ARAPFVP region (*Aurelia* sp.1: residues 538 to 544) and the NRAPYIP region (*N. vectensis*: residues 513 to 519). These regions are different from that observed in *H. sapiens* (MLAPYIP). However, this variation most likely does not affect the conserved function, because of the invariability of key functional amino acids in this region. An asparagine residue was identified in the highly conserved EVNAP region in all 7 species examined but not in the simplest animal *T. adhaerens*. The pI deviated from neutral, and the transmembrane analysis and hydrophilicity profile indicat that p*AS*HIF and p*NV*HIF are functional in the nucleus, not in the membrane or cytoplasm.

The Cnidaria includes five classes: Anthozoa (corals, sea anemones and sea pens); Hydrozoa (hydras and marine hydrozoans); Cubozoa (box jellyfish); Scyphozoa (true jellyfish); and Staurozoa (stalked jellyfish). The phylogenetic analysis suggests that HIF-1α protein potentially originated from the non-metazoan Placozoa, and then the diploblastic non-bilaterian Cnidaria separated from the triploblastic bilaterian (Fig. 4). The Cnidarian subclade includes *N. vectensis* (Anthozoa), *H. magnipapillata* (Hydrozoa) and *Aurelia* sp.1 (Scyphozoa), and the Scyphozoa and Hydrozoa are more closely related than Anthozoa. The results of the HIF-1α protein phylogenetic analysis were consistent with the conclusions of the phylogenetic analysis using the ribosomal small subunit (SSU) and mitochondrial genome structure [20]–[21]. Scyphozoa substantially deviates from Anthozoa and Hydrozoa in life history. Most medusozoans have a biphasic life cycle with alternating asexual polyp phases and sexually reproductive medusa (jellyfish) phases. In contrast, Anthozoa and Hydrozoa exclusively live on the substrate throughout life [22]–[24]. These organisms may have lost their medusa phases in the history of evolution as observed in freshwater hydras. The above description indicates that the polyps most likely preceded the medusa in Cnidarian evolution [20].

In addition to the phylogenetic analysis of the HIF-1α protein sequences, the HIF-1α functional domains from 6 species were also compared to assess HIF-1α evolution in animals (Fig. 5). No significant differences were observed in the conserved bHLH and PAS domains, but wide variations existed in the ODD and C-TAD domains. It is reasonable to suggest that the HIF-1α of *T. adhaerens* and *H. magnipapillata* most likely originated from a basic transcription factor that contained the bHLH and PAS domains [25]. For example, the non-metazoan eukaryote *Schizosaccharomyces pombe*, possess a transcription factor called Sre1 that contains a bHLH domain regulated by the predicted prolyl hydroxylase family member Ofd1 in an oxygen-dependent manner [26]. Perhaps, at some point in evolutionary history, the basic transcription factor of *T. adhaerens* or a Cnidarian ancestor accidentally obtained a proline residue that could be hydroxylated by prolyl-hydroxylase domain enzymes (PHDs) to form CODD or NODD under conditions of increased oxygen [27]. *Aurelia* sp.1 and *N. vectensis* acquired the C-TAD domain from their ancestor. However, *H. magnipapillata* retained the ancestral characteristics or lost the C-TAD domain during the evolution from sea to fresh water. An interesting finding is that the structure of p*AS*HIF is more closely related to the higher animals than the other Cnidarians; however, the *HIF-1α* gene structure analysis provides the opposite result, suggesting that *Aurelia* sp.1 belongs to the oldest eumetazoan Cnidarian (Fig. 6). The presence of a complete functional domain indicates that *Aurelia* sp.1 is the better able to regulate its activities to adapt to various oxygen environments, whereas the conserved genomic structure suggests that the genome is stable and genetic during evolution. The number and length of the *HIF-1α* gene introns significantly increased from low to high animals given the genome duplication. This phenomenon was most evident in the last exon of Placozoa and Cnidarian that contains the ODD and C-TAD domains. This exon expanded into 8 exons in *S. purpuratus* and 7 exons in *H. sapiens* [28]. This variation most likely leads to increased alternative splicing in higher animals. Although alternative splicing between exon 9 and 10 could remove the HIF-1α ODD domain of in *T. adhaerens*, our 3′RACE data and analysis of the *Aurelia* sp.1 genome sequence did not identify this mechanism. In addition, the entire last exon existed in the genome of *Aurelia* sp.1 and *N. vectensis*. The ODD and C-TAD domains were more likely to be located in one exon instead of several exons.

Post-translational hydroxylations catalysed by PHDs and FIH control the HIF protein degradation in normoxia. In other words, the HIF protein expression and stability is induced by hypoxia. The immunoblotting revealed that the p*AS*HIF existed in the polyps and medusa of *Aurelia* sp.1 (Fig. 7). The approximately 75kDa band was induced by hypoxia as determined by comparisons between the hypoxic and reference groups of the polyps and medusa with densitometry analysis. The target bands of normoxia were too weak to identify with naked eye. At the same time, the densitometry analysis indicated that the relative expression of the p*AS*HIF had significantly difference between polyps and medusa with the same conditions. Moreover, the relative expression was higher in medusa than polyps under hypoxia (Fig. 8). This result perhaps indicated that polyps needed less expression of p*AS*HIF than medusa to cope with the same hypoxic conditions, which may support the view that polyps have more hypoxic tolerance than medusa. To our knowledge, the sessile polyps of *Aurelia* sp.1 can reproduce free-swimming medusa by asexual strobilation [29]. In that process, some notable characteristics have changed in terms of size, motion and predation as well as striated and smooth muscle differentiation [30]–[31]. These variations enhanced the oxygen demands to satisfy the energy consumption required for movement and predation. Furthermore, this shift increases the oxygen-dependency of the medusa. Therefore, oxygen regulation mechanisms such as the PHD-HIF oxygen-sensing system, is necessary to adapt to variations in environmental oxygen variation in jellyfish.
